# Primary lung sebaceous carcinoma successfully treated with radiotherapy and pembrolizumab: A case report

**DOI:** 10.1111/1759-7714.14770

**Published:** 2022-12-09

**Authors:** Yasumiko Jodai, Shohei Hamada, Mikiko Yamada, Yuiko Masuda, Moriyasu Anai, Takayuki Jodai, Yusuke Tomita, Sho Saeki, Hidenori Ichiyasu, Takuro Sakagami

**Affiliations:** ^1^ Respiratory Medicine Kumamoto University Kumamoto Japan

**Keywords:** pembrolizumab, primary lung sebaceous carcinoma, radiotherapy

## Abstract

Sebaceous carcinoma is a rare cutaneous malignant tumor, usually occurring on the eyelids, head, neck, and trunk. There have been few reports about sebaceous carcinoma with primary lung cancer, for which optimal therapy has not yet been established. A 70‐year‐old man presented with a mass in the left iliac bone and tumor of the lower left lung. The morphological characteristics of the iliac bone biopsy pathology and immunostaining results showed sebaceous gland differentiation. After systemic examination, we diagnosed a primary lung sebaceous carcinoma with intrapulmonary and bone metastases. PD‐L1 was positive in 1%–24% of tumor cells, and microsatellites were stable. We detected protein kinase B (AKT1) mutations using the Oncomine Dx target test. Palliative radiotherapy (RT) of a total of 45 Gy was provided in 15 fractions to the left iliac region, which resulted in a 25% reduction in the tumor size. Subsequently, four courses of first‐line pembrolizumab led to a 30% reduction in the total tumor count. RT and pembrolizumab may be treatment options for certain rare primary sebaceous carcinomas of the lungs. A synergistic effect from RT and subsequent administration of immune checkpoint inhibitors may have contributed to tumor reduction.

## Graphical abstract

Primary sebaceous carcinoma of the lung is very rare, and no effective treatment has been yet established. We successfully treated lung sebaceous carcinoma with sequential treatment with radiotherapy and anti‐PD‐1 antibody therapy. The tumor had AKT1 mutation and was positive for PD‐L1 expression, suggesting AKT‐PD‐L1 pathway may be implicated in the response to therapy.

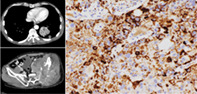

## INTRODUCTION

Sebaceous carcinoma is a rare malignant tumor of the sebaceous gland.^1^, ^2^ Extraocular sebaceous carcinoma is rare, with primary lung tumors being extremely rare.^3^, ^4^ Sebaceous carcinomas are aggressive neoplasms, and develop distant metastasis in 20%–25% of cases.^5^ Due to its rarity, there is no established treatment for sebaceous carcinoma with distant metastasis.

Herein, we present a case of primary lung sebaceous carcinoma with considerable bone metastasis, positive for PD‐L1 and protein kinase B (AKT1), that was successfully treated with radiotherapy (RT) and pembrolizumab.

## CASE REPORT

A 70‐year‐old‐man presenting with severe pain extending from his left buttock to the thigh was referred to our institute, after computed tomography (CT) revealed masses in the left lung and left iliac bone. The patient's general condition was poor, with an Eastern Cooperative Oncology Group Performance Status (PS) of 4. The patient was unable to lie supine due to the severity of the pain.

**FIGURE 1:**
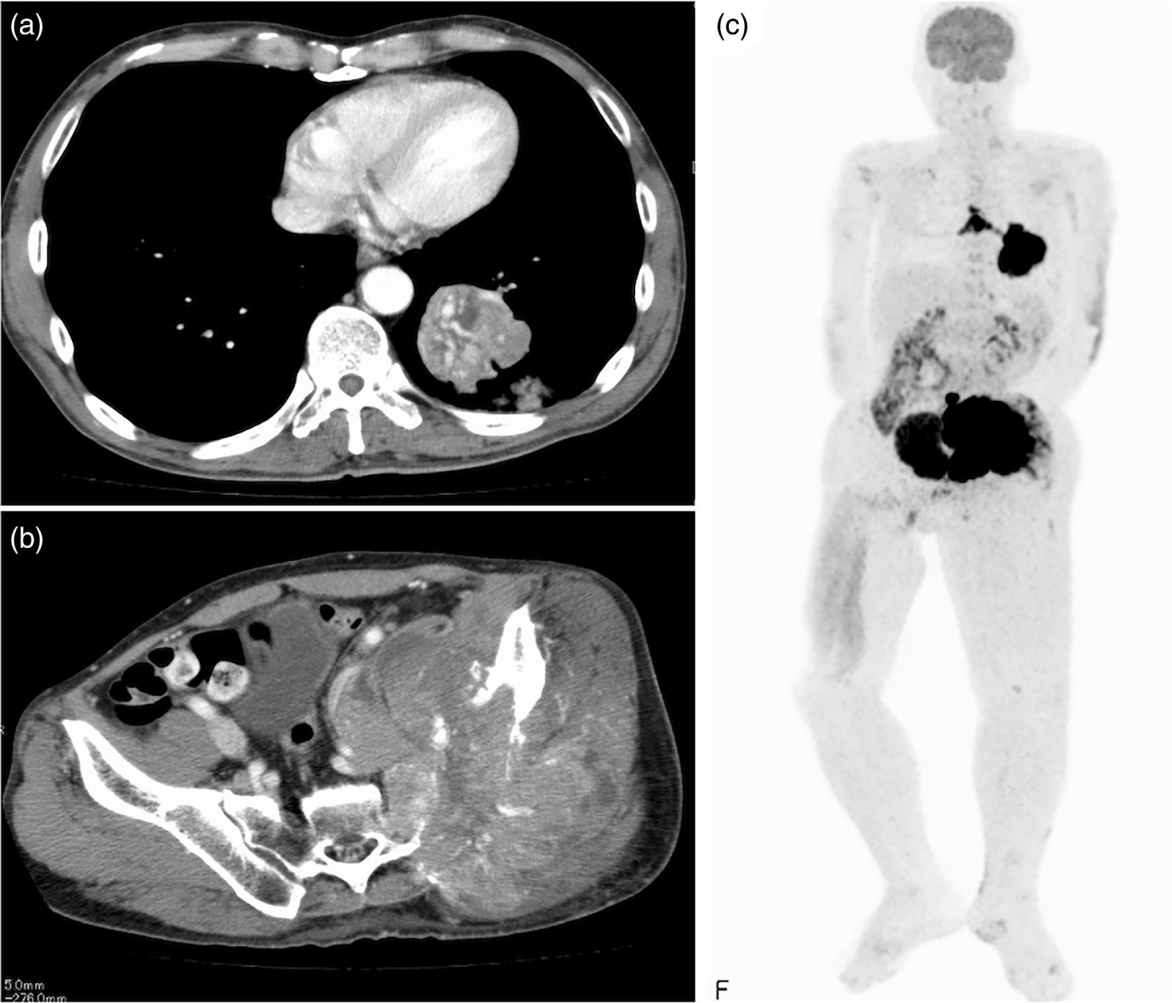
Contrast CT and PET‐CT. (a) A mass lesion 51 mm in length with an angiogram sign was observed in the lower lobe of the left lung. Peripherally to the same mass, a 47 mm long mass and a significantly enlarged ipsilateral hilar lymph node were observed. (b) A 146 mm long mass was found in the left iliac bone, extending into the pelvic cavity and muscle layer. There was no invasion into the skin or subcutaneous tissue. (c) PET showed abnormal FDG accumulation consistent with a left lung mass, mediastinal and left hilar lymph nodes, and a left iliac mass. No abnormal FDG accumulation was found in other areas. CT, computed tomography; FDG, fluorodeoxyglucose; PET‐CT, positron emission tomography.

Blood tests revealed an elevated carcinoembryonic antigen (CEA) level of 4.2 ng/ml and cytokeratin 19 fragment (CYFRA) level of 111.1 ng/ml. Elevated lactate dehydrogenase and C‐reactive protein levels, as well as anemia were also observed. Contrast‐enhanced CT revealed a mass in the lower lobe of the left lung. Peripherally, a similar mass and an enlarged ipsilateral hilar lymph node were observed (Figure 1a). A large mass was found in the left iliac bone, extending into the pelvic cavity and muscle layer (Figure 1b).

**FIGURE 2:**
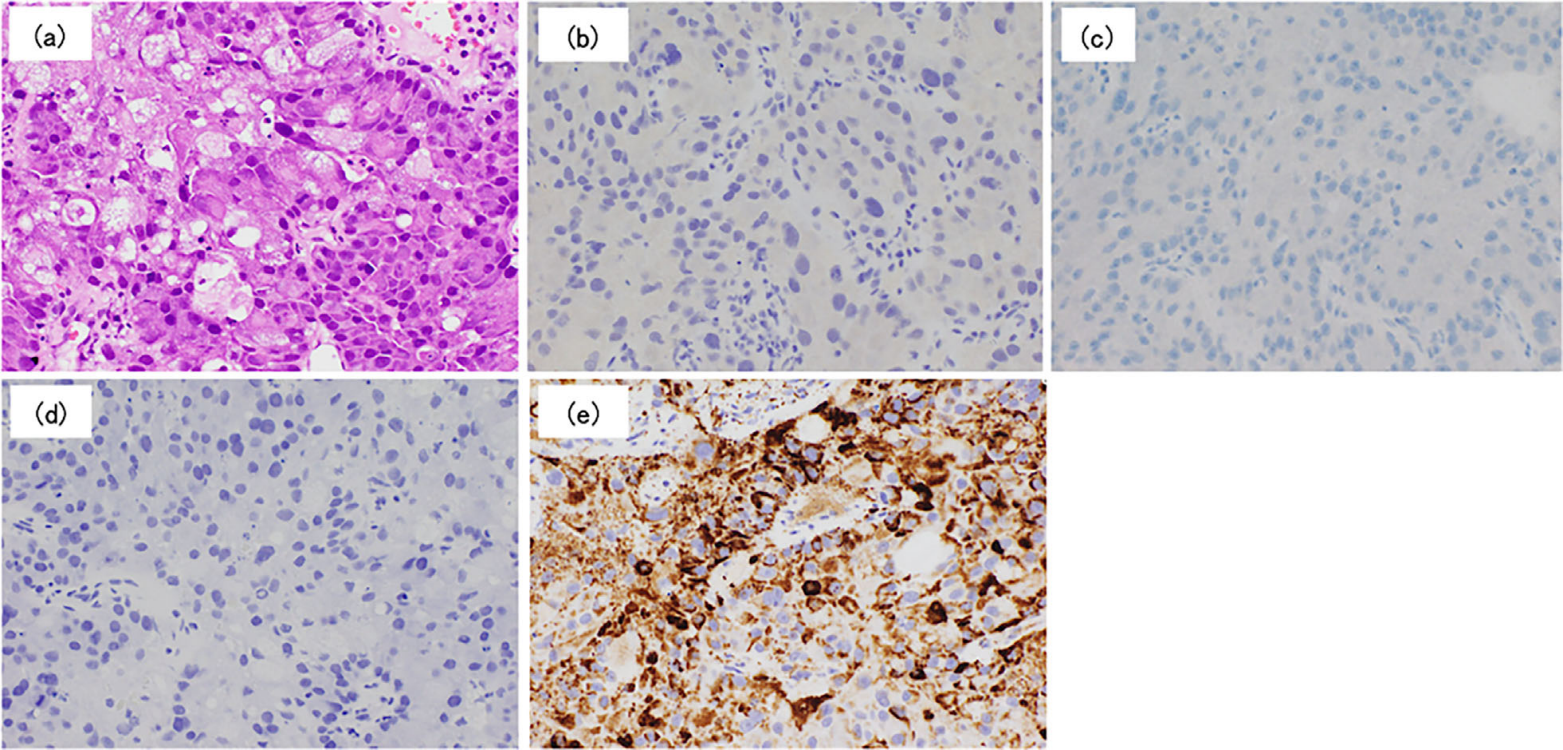
Biopsy results for the left iliac mass. (a) Metastasis of a carcinoma species showing enhanced proliferation was confirmed, and the tumor cells had foamy cytoplasm and unevenly distributed nuclei. Hematoxylin–eosin stain, original magnification x200. (b) Immunohistochemical staining was negative for thyroid transcription factor‐1 (TTF‐1) (original magnification x200), (c) and P40 was also negative (original magnification X200). (d) Gross cystic disease fluid protein‐15 (GCDFP‐15) and apocrine differentiation marker were negative (original magnification x200), as were GATA binding protein‐3 (GATA 3) and mammaglobin androgen receptor. (e) Adipophilin, a marker of sebaceous differentiation, was positive (original magnification x200).

Iliac bone biopsy revealed tumor cells with foamy cytoplasm and unevenly distributed nuclei. Immunostaining results were negative for thyroid transcription factor‐1 (TTF‐1), Napsin A, and p40. Additionally, the apocrine differentiation marker GCDFP‐15 was negative, and adipophilin, a marker of sebaceous differentiation, was positive (Figure 2).

Positron emission tomography‐CT and magnetic resonance imaging revealed no other neoplastic lesions (Figure 1c), and this lesion was diagnosed as a primary sebaceous carcinoma of the lung with ipsilateral intrapulmonary and bone metastases. The TNM classification was T3N2M1b and clinical stage was IVA. PD‐L1 expression was positive in 1%–24% of tumor cells and the microsatellites were stable. AKT1 mutations were detected using the Oncomine Dx target test (OD × TT).

**FIGURE 3:**
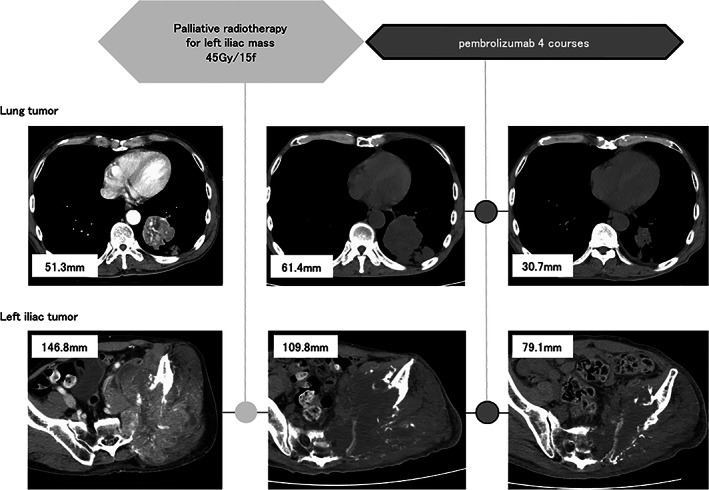
Treatment course. The patient received palliative radiotherapy of 3 Gy per session for a total of 45 Gy to the left iliac tumor for the purpose of pain relief. After radiotherapy, the iliac tumor reduced in size by 25%, from 146.8 to 109.8 mm, while the lung mass tended to increase in size. After the completion of in situ radiotherapy, pembrolizumab was started. Following four courses of pembrolizumab, the primary lung lesion was reduced by 50% from 61.4 to 30.7 mm, and the total tumor size reduced by 30%.

Initially, the patient received a palliative RT of total of 45 Gy to the left iliac bone, after which the bone tumor mass was reduced by 25% after RT. During RT, the primary lung tumor increased in size by 19.6% and total tumor count increased by 2.5%. After RT, pembrolizumab was administered as the first‐line therapy. After four courses of pembrolizumab, the primary lung tumor, bone tumor, and total tumor counts were reduced by 38, 46, and 30%, respectively (Figure 3). The effectiveness determination based on Response Evaluation Criteria in Solid Tumors (RECIST) was partial response. Tumor markers CEA and CYFRA decreased to 2.8 and 7.5 ng/ml, respectively. The scale of performance status was improved to 1 and the patient was able to continue treatment.

## DISCUSSION

We diagnosed an extremely rare case of primary sebaceous carcinoma of the lung, which was AKT1 mutation positive, PD‐L1 positive, and microsatellite stable. The patient responded to sequential treatment with RT and pembrolizumab. Only two cases of primary sebaceous carcinoma of the lung have been previously reported; clinical characteristics of those cases and our case are shown in Table 1. Surgery was performed in the previously reported cases based on the clinical stage of tumor. Both cases 1 and 2 maintained recurrence‐free survival during the observational period.^3^, ^4^

**TABLE 1:** Patient characteristics of previously reported cases and our case

	Age	Sex	Clinical stage	Histopathology resulting in a diagnosis	Therapy	Outcomes (observational period)
Case 1 (Borczuk et al.)	78	Man	unknown (tumor size was 2.2 cm without distal metastasis.)	Mucin, Oil red O staining	Surgery only	No recurrence (3 years after surgery)
Case 2 (Hanaka et al.)	75	Male	IIA	Oil red O staining	Surgery only	No recurrence (5 months after surgery)
Our case	70	Male	IVB	Adipophilin	Palliative RT, ICI	Partial response (6 months)

Abbreviations: ICI, immune checkpoint inhibitor; RT, radiotherapy.

The histopathological pattern of extraocular sebaceous carcinoma is an infiltrating, homogeneous nested/lobular vacuolated sebaceous neoplasm of the dermis, characterized by foamy, scalloped nuclei, atypia, and mitosis of malignant cells.^6^

The pathology of our case had morphological features such as clear foamy cytoplasm, which is a characteristic of sebaceous carcinoma. No histological findings were suggestive of a common lung carcinoma although squamous cell carcinoma of the lung with sebaceous gland differentiation has previously been reported.^3^ Furthermore, since only adipophilin was positive on immunostaining and there were no intraorbital or skin lesions, which is the most common primary extraorbital lesion, the patient was diagnosed with primary lung sebaceous carcinoma.

Few case reports have been published on PD‐1 inhibitors for sebaceous carcinomas with metastasis with complete response (CR) or near‐CR efficacy.^7^, ^8^ Hence, there is limited evidence on immunotherapy in patients with primary sebaceous carcinoma of the lung.

Here, sequential administration of immune checkpoint inhibitors (ICIs) after palliative RT was successful. The result may have been a synergistic effect of RT and ICI. Formenti et al. suggested that ionizing radiation to a tumor‐encompassing target produces effects beyond cell death, sending specific signals to the host immune system.^9^ They further suggested that irradiation at doses sufficient to directly cause immunogenic cell death triggers specific danger signals. These cues are then sensed by immune components, such as dendritic cells, activating an adaptive immune response. In cancer RT, this process may promote antitumor immunity. The upregulation of checkpoint proteins after RT indicates a link between RT and ICIs.^10^ The synergistic effect of RT and ICIs on locally advanced or metastatic non‐small cell lung cancer has been studied in clinical trials and has been demonstrated to improve survival.^10^, ^11^ In our case, preceding RT may have promoted antitumor immunity and ICI response.

Here, AKT1 mutations were detected using the ODxTT. No other cases of AKT1 mutation positivity in sebaceous carcinomas have been reported. The AKT/extracellular signal‐regulated kinase signaling pathway has been associated with PD‐L1 expression^12^; thus, AKT1 mutations may impact RT and anti‐PD‐1 antibody therapy efficacy.

In conclusion, we successfully treated an extremely rare primary sebaceous carcinoma of the lung with sequential RT and anti‐PD‐1 antibody therapy. The tumor had an AKT1 mutation and expressed PD‐L1, suggesting that the AKT‐PD‐L1 pathway may influence the response to therapy. A treatment strategy combining ICI with RT may induce synergistic effects and be a treatment option for sebaceous carcinoma.

## AUTHOR CONTRIBUTION

YJ drafted the original manuscript. SH and YT reviewed the manuscript draft and revised it critically on intellectual content. MY, YM, MA, TJ, SS, HI and TS reviewed the manuscript draft. All authors approved the final version of the manuscript to be published.

## CONFLICT OF INTEREST

All authors declare no potential conflict of interest.
